# Does Hydrotherapy Impact Behaviours Related to Mental Health and Well-Being for Children with Autism Spectrum Disorder? A Randomised Crossover-Controlled Pilot Trial

**DOI:** 10.3390/ijerph17020558

**Published:** 2020-01-15

**Authors:** Whitney Mills, Nicholas Kondakis, Robin Orr, Michael Warburton, Nikki Milne

**Affiliations:** 1 Physiotherapy Department, Bond University Faculty of Health Sciences and Medicine, 2 Promethean Way, Robina, QLD 4226, Australia; whitlmills@gmail.com (W.M.); kondi_junior@hotmail.com (N.K.); rorr@bond.edu.au (R.O.); 2 Physiotherapy Department, Gateway Physiotherapy, 17/590 Mount Gravatt Capalaba Road, Wishart, QLD 4122, Australia; physio4u@bigpond.net.au

**Keywords:** Autism Spectrum Disorder, behaviours, hydrotherapy, physiotherapy, Child Behaviour Checklist

## Abstract

Background: Children diagnosed with Autism Spectrum Disorder (ASD) are less physically active than typically developing children due to reduced socialisation and delayed gross-motor skills, negatively impacting social, emotional and physical well-being. This study aimed to determine whether hydrotherapy influences behaviours which impact mental health and well-being in children with ASD. Methods: A within-subjects, randomised crossover-controlled pilot trial was used over 8 weeks. Children aged 6–12 years and diagnosed with ASD (n = 8) were randomly allocated to Group 1 (n = 4) or Group 2 (n = 4). All children participated in hydrotherapy intervention from either weeks 1 to 4 or weeks 5 to 8. The Child Behaviour Checklist (CBCL) measured behaviour changes impacting mental health and well-being, administered at weeks 0, 4 and 8. Results: No observable differences were found in CBCL subscales between Group 1 or 2 at baseline (week 0). Paired-samples t-tests revealed significant improvements post-intervention: Anxious/Depressed subdomain (*p* = 0.02) and the Internalising Problems Domain Summary (*p* = 0.026), with large effect size (d = 1.03 and d = 1.06 respectively). Thought Problems (*p* = 0.03) and Attention Problems (*p* = 0.01) both significantly improved post-intervention. The Total Problems score significantly improved post-intervention (*p* = 0.018) with a large effect size (d = 1.04). Conclusion: Hydrotherapy may enhance behaviours impacting mental health and well-being of children with ASD and could be considered a beneficial therapy option.

## 1. Introduction

According to the Diagnostic and Statistical Manual of Mental Disorders (DSM-5), Autism Spectrum Disorder (ASD) is a neurodevelopmental disorder defined by deficits in social interaction skills, communication (verbal and non-verbal), and restricted repetitive patterns of behaviour [1]. Research has suggested that children with ASD have delayed gross motor development [2] and, consequently, reduced participation in physical activity in comparison to typically developing children [3]. This reduced participation in physical activity has implications for the physical health of children with ASD, as a sedentary lifestyle can lead to cardiovascular disease, obesity, type-II diabetes, and other related health issues [4]. Not only can a child’s physical health be compromised by physical inactivity, but so can their mental health—both of which have implications on a child’s overall well-being [5]. 

Well-being has been previously defined as an individual’s overall state of health, including physical, psychological, social and emotional health [6]. Improved well-being promotes a better self-image, positive interactions with family and peers, and a greater sense of happiness [7]. The effects of physical activity have been extensively researched in typically developing children, showing reduced levels of anxiety and depression, improved self-perception and academic performance [8,9]. Similar benefits have been proposed for children with ASD, indicating they too may benefit from physical activity [10,11]. 

In the past, a variety of funded therapeutic options have been available for children with ASD, including speech and language therapy, behavioural interventions, and occupational therapy. However, these treatment options do not specifically focus on physical activity and fail to address gross motor skill and coordination deficits, which are prevalent in this population [2]. Without proficient gross motor skills, the ability for children with ASD to participate in physical activity may be reduced [12]. This may be particularly evident in the school environment, where running, jumping, hopping, kicking, throwing and catching are just some of the necessary gross motor skills required to participate in play and games with peers. A child’s inability to participate in these activities may lead to social isolation which, consequently, could further reduce mental health and well-being [5].

Hydrotherapy (or water-based activity) is an environment that may be conducive to encouraging physical activity in children with ASD. The buoyancy of water can assist with movement, balance, and coordination [13]. Water also provides an opportunity for social interaction through aquatic games and activities [14]. Hydrotherapy may include aquatic programs, water-based activities or swimming programs. A previous systematic review by Mortimer, Privopolous and Kumar in 2014 [15] revealed four previous studies that had explored the effectiveness of hydrotherapy in the treatment of social and behavioural aspects of children with ASD. However, they concluded that the literature was limited by a lack of controlled trials and standardised outcome measures to evaluate the effects. This pilot study aims to address this concern and to contribute further to this field of empirical research literature. Therefore, this study aims to determine whether hydrotherapy (or water-based therapy) influences behaviours related to the mental health and well-being of children with ASD. Specifically, the study aims to explore the effects of hydrotherapy on (i) emotional behaviours related to the domain of internalising problems (i.e., syndromes of anxious/depressed, withdrawn/depressed and somatic complaints); (ii) emotional behaviours related to the domain of externalising problems (i.e., syndromes of rule breaking behaviour and aggressive behaviour) and; (iii) behaviours impacting social functioning (i.e., syndromes of social problems, thought problems and attention problems). 

## 2. Materials and Methods 

### 2.1. Participants

Children with ASD were recruited from Gateway Physiotherapy, local general practitioners, Autism QLD Facebook page, and notice board at a local Christian College. All participants were aged 6–12 years (mean = 8.72 years ± 1.99 years). One child had additional medical diagnoses; connective tissue disorder, slow brain waves, heart murmur, and chromosomal deletion. One child was also diagnosed with an intellectual disability. Five children were receiving regular medications. One child was taking Lovan and Vyvanse. Two children were taking stimulants (dexamphetamine/Ritalin). One child was taking Circadin and another was taking Melatonin. See Table 1 for participant characteristics. The inclusion criteria for this study included children who were (a) a Gateway Physiotherapy client and/or a patient of a local general practitioner; (b) aged 6–12 years, and; (c) had previously been diagnosed with Autism Spectrum Disorder from a medical doctor or specialist. Children were excluded from this study if they had (a) contraindications to hydrotherapy as outlined in the Screening for Contraindications/Precautions to Hydrotherapy form used for this research study or; (b) a significant fear of water. 

### 2.2. Study Design

Using a sample of convenience, participant recruitment began November 2016 and continued until participant and guardian information sessions were held 23 January 2017–6 February 2017 by the research team. Information sessions included participant baseline assessments, gathering general participant information, and providing each participant and their guardian with a study information sheet outlining the aim and methods of the intervention study. Both parents/guardians and participants then gave informed, voluntary written consent for their child to participate in the research study. Ethical approval for this study was obtained from the Bond University Human Research Ethics Committee (BUHREC) (15409) prior to commencing the research project, and the work was carried out in accordance with the BUHREC ethical standards of human experimentation and with the Declaration of Helsinki as revised in 2000 [16]. This study was considered by the BUHREC to be a non-clinical trial as it was limited to evaluating outcomes of an already established health care (physiotherapy) management option (hydrotherapy) for children with ASD and for this reason this study was not registered as a clinical trial. However, this study was compliant with the National Statement on Ethical Conduct in Human Research in Australia [17] at the time the study was ethically approved (November 2015). 

A within-subjects, randomised crossover-controlled pilot study design was used, where participants acted as their own control, a process previously reported in the literature for investigating hydrotherapy interventions [18]. No changes were made to the methods after the study commenced. Participants were randomly allocated to either group 1 (G1) or group 2 (G2) using a randomisation function in Microsoft Excel. Both groups experienced four weeks of hydrotherapy and four weeks with no hydrotherapy (control period). G1 participated in hydrotherapy sessions from weeks 1 to 4, and G2 participated in hydrotherapy sessions from weeks 5 to 8. For the duration of the 8 weeks all participants continued with their regular therapies and/or physical activity programs. Any additional therapies received by the participants during the study were recorded. The participants were not blinded to the aims of this study or to their group allocation due to the nature of its design.

### 2.3. Hydrotherapy Intervention

The hydrotherapy interventions were conducted during the months of February to March 2017 at the Mt Gravatt East hydrotherapy pool in Brisbane, Queensland, Australia. The hydrotherapy sessions lasted 45 minutes and were planned once weekly. Several children required make-up hydrotherapy sessions due to illness or weather conditions (which occurred individually, rather than in a group) and as a result received between 0 and 2 hydrotherapy sessions for select weeks during the study but averaging one session per week over four weeks. Session times were arranged in negotiation with parents and kept as consistent as possible to create routine for the children. Regular hydrotherapy sessions were scheduled every Friday commencing between 15:30 and 15:50. 

Each session took place in either an indoor pool sized 20 m × 10 m × 1.2 m with 31.9 ± 0.8 °C water temperature or an outdoor pool sized 25 m × 20 m × 1.0–1.5 m with 29.4 ± 1.1 °C water temperature. Throughout the hydrotherapy sessions, air temperature was 30.1 ± 2.1 °C and humidity was 57% ± 19%. All sessions were led by one of two student physiotherapists (N.K. or W.M.), a registered physiotherapist (M.W.), and one exercise physiologist employed with Gateway Physiotherapy. All sessions were offered in small groups with a 1:1 ratio of instructor to child with the exception of week 1, where three instructors worked between four children. Parents were able to observe the hydrotherapy sessions if they desired. All participants in G1 and G2 had similar hydrotherapy programs. 

Each session included a 0–5 minute warm up (using cardiovascular fitness activities) and cool down (using relaxation and sensory input activities), with a variety of activities targeting, swimming skills, balance, eye-hand coordination, and cognitive tasks used in between the warm up and cool down. All activities and equipment used during the hydrotherapy sessions are outlined in Table 2. In attempts to tailor hydrotherapy sessions to each child’s abilities and interests, the middle component of hydrotherapy sessions was executed according to the child’s ability and age. Each hydrotherapy session included activities from a communal list of activities (see Table 2) which were designed by a physiotherapist who had more than 15 years of experience undertaking pediatric hydrotherapy sessions with children of varying physical and mental capabilities. In order to keep the children engaged, a play-based approach to therapy was implemented, where the duration and order that each activity was performed varied for each child based on their interest and motivation on the day. In each group session, the children had the opportunity to interact socially with their instructor and peers during the play-based therapy.

### 2.4. Outcome Measures

The Child Behaviour Checklist (CBCL) [19] is a short, standardised questionnaire, which aims to identify social, behavioural and emotional problems. Parents were asked to complete the CBCL at weeks 0, 4 and 8 to determine whether the addition of hydrotherapy sessions resulted in participant behavioural changes during the study. This schedule adhered to the Achenbach System of Empirically Based Assessment (ASEBA) recommendation to allow at least one month between assessments [20]. This approach minimises test–retest attenuation and allows adequate time for behavioural changes to occur and become evident [21]. 

The questionnaire includes eight subscales or ‘syndromes’: Anxious/Depressed, Withdrawn/Depressed, Somatic Complaints, Social Problems, Thought Problems, Attention Problems, Rule Breaking Behaviour, and Aggressive Behaviour. These syndromes are divided amongst three domains: Internalizing Problems, Externalizing Problems, and Other Problems. The syndromes cover a range of concepts associated with children’s behaviours and have been used previously to assess children’s mental health and well-being status [22,23,24].

The CBCL has been validated for use with children with ASD, with a high test–retest reliability (Pearson’s r = 0.88-0.9) and moderate inter-rater reliability (Pearson’s r = 0.73–0.76) [20,21]. It has a moderate-to-high internal consistency (Cronbach’s alpha = 0.63–0.8) [20,21], indicating the individual syndromes are moderate-to-highly related to the other syndromes within the same domain. The CBCL also has high sensitivity (0.92) and high specificity (0.82) [21]. Lastly, the CBCL has good content and criterion validity, demonstrating its ability to significantly discriminate (*p* < 0.01) between children with and without clinical level scores [20].

Verbal information was provided to parents informing them how to complete the CBCL. In attempts to avoid inter-rater reliability issues, the same parent was requested to complete the CBCL at weeks 0, 4 and 8. To minimize the risk of influencing the results, the researchers were blinded to the results of the participant outcome measures. This was achieved by the parents of participants placing the CBCL in a sealed envelope, which was given to a fourth researcher (RO) who was not in contact with the participants nor knew the participant group allocations. 

The enjoyment scale, previously described by Jelsma and colleagues [25] was used throughout the hydrotherapy sessions to monitor the level of comfort, enjoyment, and participation willingness of study participants during their therapy activities. The enjoyment scale is a 5-point visual scale (0 to 4), where participants point to a score at the end of the hydrotherapy sessions. The higher the score indicated by the child, the more fun the child reported they had during the intervention. The scale helped ensure the children were not becoming distressed or uncomfortable by the unfamiliar hydrotherapy sessions.

### 2.5. Statistical Analysis

Data obtained from the CBCL forms were manually entered into the ASEBA Web application in a de-identified form. T scores from the ASEBA-Web application were derived by first obtaining z scores for the raw CBCL scores, using the following formula [26]:

$$z=\frac{x-M}{\mathrm{SD}}$$

where x is the raw CBCL scores, M is the mean of the normative sample, and SD is the standard deviation of the normative sample derived from national and international normative data. T scores have a mean of 50 and a standard deviation of 10 [27]. With these integers, T scores were then calculated using the following formula [26]:

T=10xz+50

where 10 is the T score standard deviation, z is the calculated z score, and 50 is the mean. Data were then exported from ASEBA-Web [19] into Microsoft Excel and IBM SPSS Statistics application for statistical analysis. 

Paired t-tests were used to examine the effect of the intervention period and control period on the combined G1 and G2 by comparing mean t scores pre- and post-intervention, and pre- and post-control periods. The paired t-tests were undertaken for all domains and syndromes of the CBCL. Paired t-tests were also used to explore differences in change scores for the intervention and control periods, using the mean intervention score change and the mean control period score change for all participants combined. The paired t-tests were applied to all domains and syndromes. Effect sizes (d), where the difference between the means was divided by the pooled SD [28], were also calculated for the within group comparisons, whereby 0.20 was considered a small effect, 0.50 was considered a medium effect and 0.80 a large effect [28]. 

## 3. Results

### 3.1. Participant Flow

**Figure 1:**
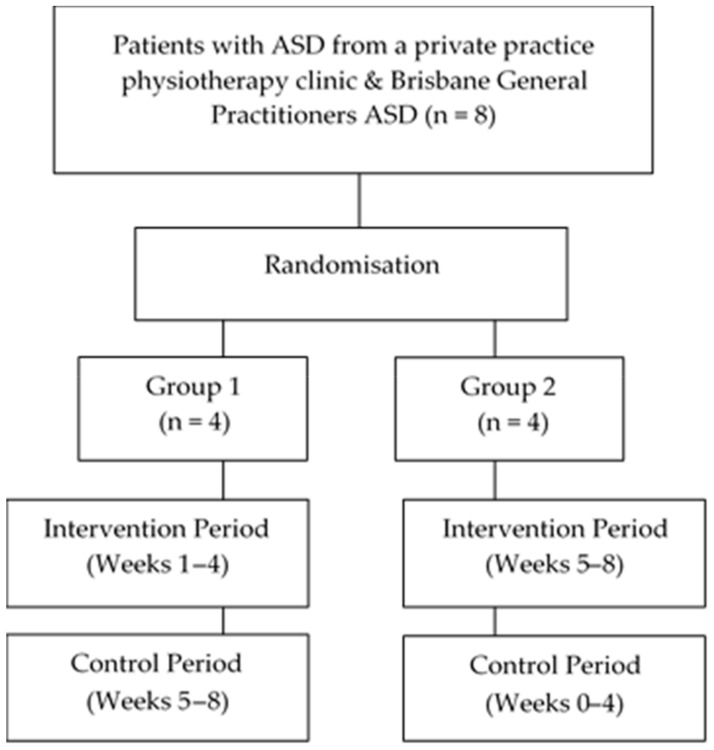
Flow of patients throughout the study.

Patient flow through the study is outlined in Figure 1. Eight children (F = 2, M = 6; mean age = 8.72 years ± 1.99 years: range 6.75–12.6 years) were included in this study with no volunteering participants meeting the exclusion criteria. All children were screened, met the inclusion criteria, and with their parents provided consented to participate in the research study. All eight children completed the hydrotherapy study.

### 3.2. Treatment Characteristics

Overall, hydrotherapy group session attendance was 93.75% and 81.25% for group 1 and 2 respectively. However, all children received their full intervention load of four sessions across four weeks with individual make-up sessions accounting for one session in G1 and three sessions in G2. Table 3 illustrates the children’s enjoyment during the hydrotherapy group interventions over the 8 weeks using the enjoyment scale. Five participants also attended additional therapies throughout the duration of the study. Given that participants acted as their own controls, any additional therapies are not expected to have affected the study results.

### 3.3. Adverse Events

There was one incident of a child excreting bodily fluids into the swimming pool, which was promptly sanitised by swimming pool attendants. Also, one child experienced bruising surrounding bilateral knees following the first hydrotherapy intervention. This was deemed to be due to an underlying connective tissue disorder and resolved quickly with no further issues. Aside from this brief event, there were no concerns or adverse effects voiced by the participants or their parents following the hydrotherapy interventions. No other serious events occurred throughout the research study. 

### 3.4. CBCL Scores

Observed mean scores suggested no noteworthy differences between group 1 and 2 at baseline (week 0) for the percentile scores in the domains listed on the CBCL (see Table 4). Paired samples t-tests between pre intervention scores (G1—week 0 and G2—week 4) and post-intervention scores (G1—week 4 and G2—week 8) revealed significant changes in scores for several domains during the intervention period (see Table 5). Significant improvements were observed in the Anxious/Depressed subdomain (*p* = 0.02) and the Domain Summary (*p* = 0.026), both with a large effect size (d = 1.03 and d = 1.06 respectively) for the Internalising Domain. For the other problems sub domains, Thought Problems (*p* = 0.03) and Attention Problems (*p* = 0.01) both significantly improved pre- to post-intervention with effect sizes ranging from large (d = 0.82) to medium (d = 0.82) respectively. The Externalizing Problems Domain Summary approached significance (*p* = 0.06) following the intervention period (see Table 5). Overall, the Total Problems score was found to be significantly improved post-intervention (*p* = 0.018) with a large effect size (d = 1.04). Additionally, pre control scores (G1—week 4 and G2—week 0) were compared to post-control scores (G1—week 8 and G2—week 4) to examine the control period effect. The paired samples t-tests indicated there was no significant change in scores during the control period.

**Figure 2:**
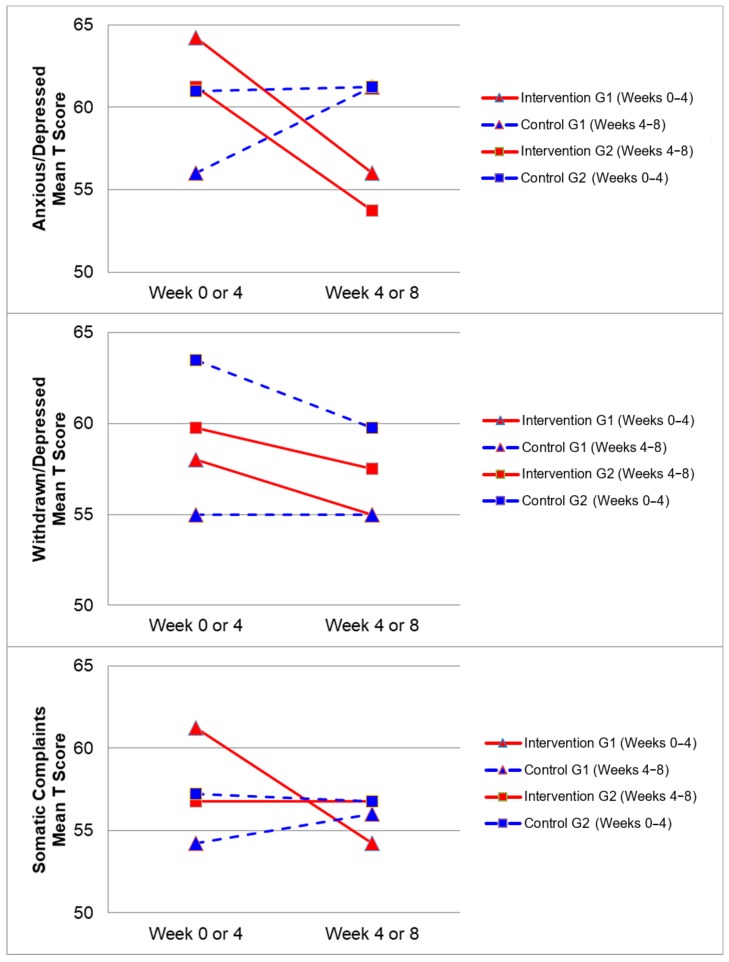
Mean t score changes within Group 1 (G1) and Group 2 (G2) based on intervention vs. control periods for anxious/depressed, withdrawn/depressed, and somatic complaints syndromes.

**Figure 3:**
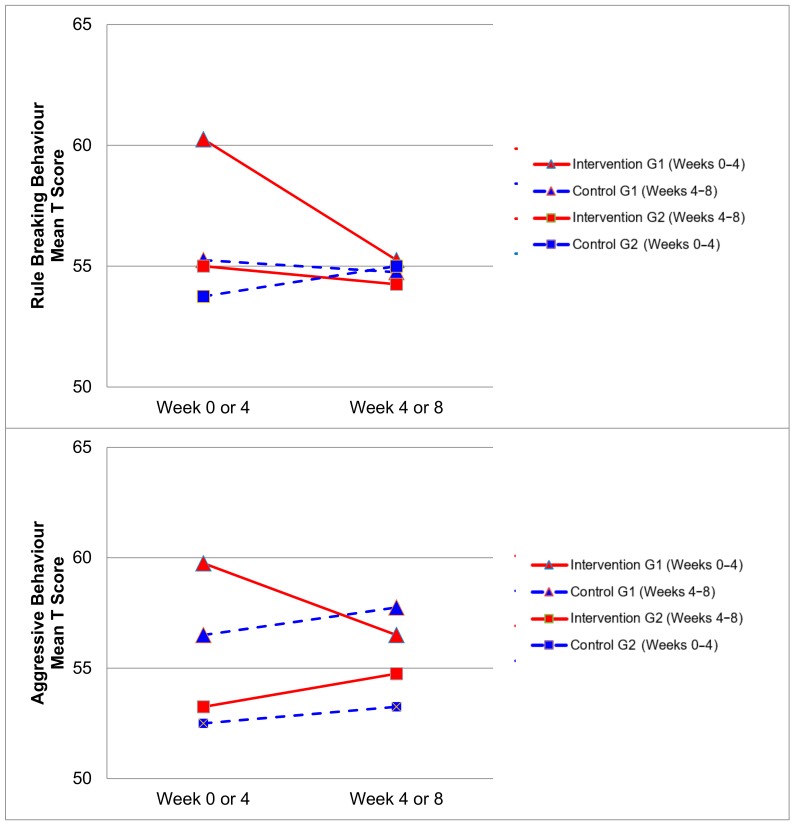
Mean t score changes within Group 1 (G1) and Group 2 (G2) based on intervention vs. control periods for rule breaking behaviour and aggressive behaviour syndromes.

**Figure 4:**
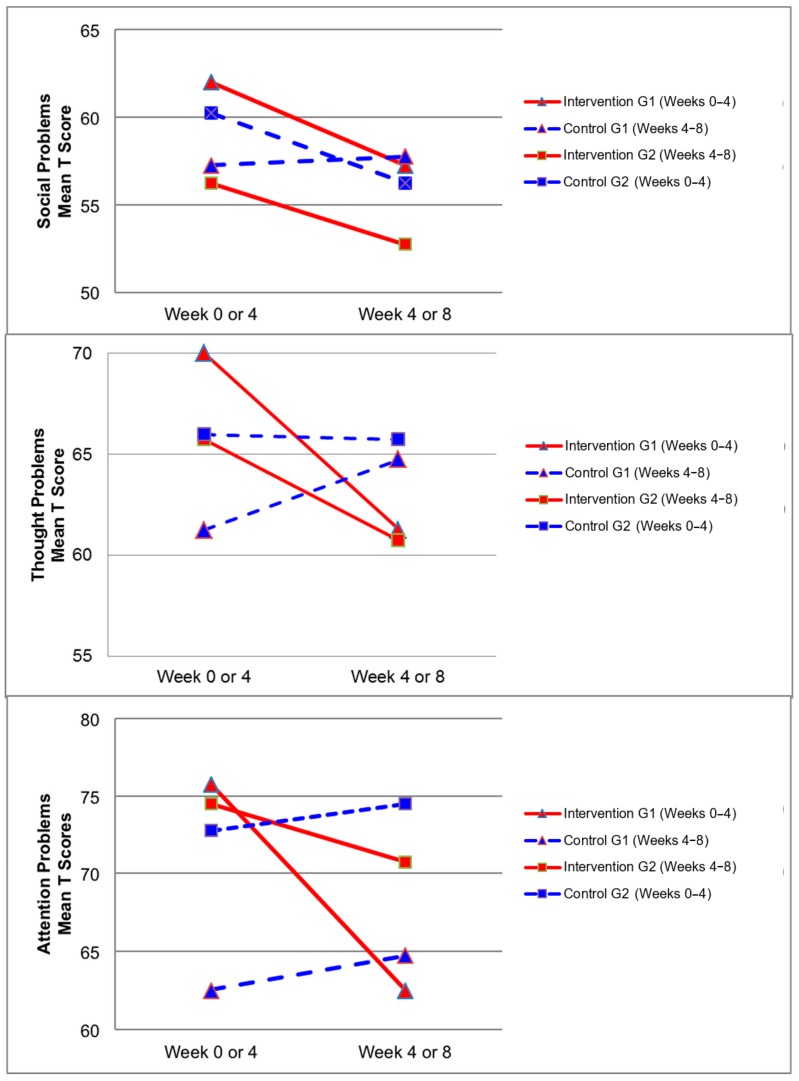
Mean t score changes within Group 1 (G1) and Group 2 (G2) based on intervention vs. control periods for social problems, thought problems, and attention problems syndromes.

**Figure 5:**
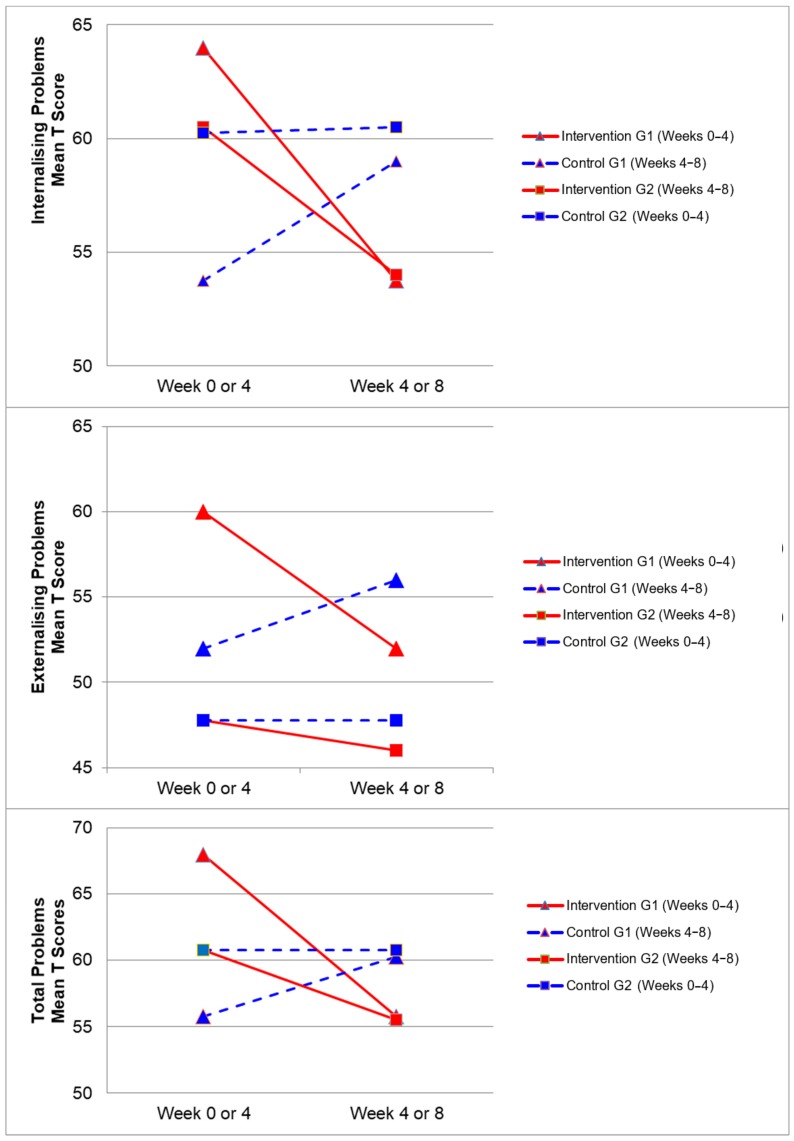
Mean t score changes within Group 1 (G1) and Group 2 (G2) based on intervention vs. control periods for the internalizing problems, externalizing problems, and total problems.

Figure 2, Figure 3, Figure 4 and Figure 5 demonstrate the intervention and control period outcomes for each of the domains and syndromes, using a mean t score for comparison. A reduction in the domain and syndrome t-score value between the two points (weeks 0 or 4 to week 4 or 8) indicates an improvement in the particular domain or syndrome, while an increase indicates a worsening of the domain or syndrome. All three domains displayed a reduction in t score values for both intervention groups (see Figure 5). G1 and G2 exhibited reductions in all syndrome t scores following the intervention period, with the exception of G1 for the Aggressive Behaviour and Somatic Complaints Syndromes (see Figure 2, Figure 3 and Figure 4). G1 also maintained improvements in the Withdrawn/Depressed and Rule Breaking Syndromes over the control period (see Figure 2 and Figure 3).

## 4. Discussion

This randomised crossover-controlled pilot trial aimed to explore the effects of a 4 week hydrotherapy (water-based) program on behaviours impacting the mental health and well-being of children with ASD. The major findings of the present study demonstrated that children with ASD may benefit from a hydrotherapy program to improve their internalising behaviours, specifically anxious and depressed behaviours, as well as reducing thought and attention problems. 

Significant improvements were observed over the intervention period for the Anxious/Depressed, Thought Problems and Attention Problems syndromes for G1 and G2 combined. No differences were observed between G1 and G2 at baseline (week 0) for all CBCL domains and syndromes nor were any significant changes found over the control period. This indicates that these significant improvements in behaviours impacting mental health and well-being may be due to the hydrotherapy sessions carried out during the intervention periods, however it is possible that a number of confounding variables may have impacted the results and these are discussed further in the limitations section below.

The CBCL syndromes that are reported in the empirical literature to be most predominately elevated for children with ASD are Social Problems, Thought Problems, and Attention Problems [29,30,31]. In the present study, significant decreases were observed over the intervention period (see Table 5) for the Thought Problems Syndrome (*p* = 0.03) and Attention Problems Syndrome (*p* = 0.01), with Social Problems Syndrome approaching significant levels (*p* = 0.08). These significant and near significant improvements in the CBCL syndromes which are most characteristic of children with ASD, indicate the potential for hydrotherapy to improve the syndromes most commonly associated with ASD.

The results of the present study relate to previously published findings from studies examining the effects of physical activity in typically developing children. A meta-analysis conducted by Ahn and Fedewa in 2011 [8], showed exercise had small to moderate effects in reducing depression, anxiety, psychological stress, and emotional disturbances in children ranging from ages 3–18 years. These outcomes are mirrored in a cohort study conducted by Galper and colleagues in 2006 [32], examining the relationship between physical activity and mental health in men and women aged 20-88 years. They discovered higher levels of habitual physical activity were associated with lower symptoms of depression and greater emotional well-being. Hydrotherapy has traditionally been under-utilised as a treatment modality in previously reported literature, compared to dry land activities, when exploring the effects of physical activity on mental health and well-being. However, there has been a recent increase in research on the effects of hydrotherapy programs for people with disabilities, and particularly children with ASD [15].

Although there were no significant results to suggest specific improvements in social well-being, anecdotal evidence received from parents indicated a noticeable improvement. One parent mentioned her child being ‘more sociable’, seeking company from family and instigating friendly physical play with another child. Multiple parents reported their children being “more relaxed” and ‘less agitated’ following hydrotherapy interventions. This was reported to the therapists to have led to a better home environment and less strained parent–child relationship. 

Improvements from hydrotherapy were well maintained over four weeks in the Withdrawn/Depressed and Rule Breaking syndromes for G1. Any gains in the remaining CBCL syndromes were not maintained for G1, and either returned to or approached baseline levels (see Figure 2 and Figure 3). This result indicates a small carry over effect was found for select CBCL domains in this study. A similar study observed the effects of aquatic exercise on sleep in children with ASD [33]. A carry over effect was observed in total sleep hours and sleep latency 4 weeks after the intervention period had concluded. Similarly, Pan and colleagues in 2010 [34] showed that improvements of aquatic skills in children with ASD were maintained for 10 weeks after a water exercise swimming program. Therefore, certain benefits of hydrotherapy may extend beyond the period of hydrotherapy treatment, although this study did not investigate these findings over a prolonged follow-up period. Whilst the results of this study indicate an immediate improvement in some behaviours impacting mental health and well-being, the lack of carry over beyond the intervention period for some syndromes may be related to the dose of hydrotherapy input over the intervention period with only one session per week. Future research in this area could consider increasing the frequency of hydrotherapy sessions over a similar period and examining the immediate and carry over effects. However, the real-world viability for families to attend repeated hydrotherapy sessions in addition to other commonly utilised therapies for children with ASD should be remain a critical consideration. 

### 4.1. Strengths

The present study had a high participation rate, with an 87.25% overall group attendance (100% combined attendance when factoring in make-up sessions) from participants and yielded significant results, demonstrating improvements in several aspects of mental health and well-being. This study has presented hydrotherapy as an enjoyable physical activity for children with ASD, which may improve behaviours impacting their mental health and well-being.

### 4.2. Limitations and Future Research

This pilot study had several limitations and the results should be considered with caution. The statistical power of this study could have been improved by increasing the sample size. Likewise, the limited number of intervention sessions in this study may have affected the statistical strength and limited the intervention effect. Many recent hydrotherapy intervention studies investigating effects on children with ASD have employed 2–3 interventions per week for 10 or more weeks [34,35,36,37,38]. Extending the intervention period and/or increasing the frequency of hydrotherapy sessions may be an advantageous addition to future studies, with the potential to magnify the intervention effects and control period carry over seen in this study. Additionally, due to the limited intervention period, this study did not explore the effects of the hydrotherapy sessions on physical activity or motor skills of the children involved and future research could consider this domain of health. 

Another limitation was the use of the enjoyment scale during the hydrotherapy interventions. A child’s score on the scale did not always coincide with observations made by therapists and parents. This was particularly evident with children who were non-verbal and exhibited more severe symptoms of ASD. These children often chose a low score, but were observed to be smiling, laughing, and enjoying themselves throughout the sessions. It is possible that some children preferred their hydrotherapy interactions with one instructor more than another on an alternate therapy day and thus the child’s responses may be reflective of their social interactions with the instructor or their peers on the day, rather than their enjoyment levels of the task. Future studies may benefit from utilising alternative methods of assessing a child’s enjoyment during therapeutic activities. Alternatively, trialing the enjoyment scale in other areas of the child’s life may be beneficial. Incorporating the enjoyment scale into activities of varying enjoyment levels and observing the child’s scores and reactions may increase the validity of this tool in the hydrotherapy setting for children with ASD, and this warrants further research. A further important limitation was our inability to guarantee that our participant cohort was representative of the wider population of children with ASD, considering that 30% of our study population had additional comorbidities and this should be considered when interpreting the results of our pilot study. Future studies beyond this pilot trial are necessary to replicate this work with larger samples. Further prospective studies could consider a using a control group, matching participants in the intervention group by age, gender, cognition and comorbidities (e.g., ADHD), controlling for variables such as individual versus group intervention ratios and should consider a longer-term follow-up assessment beyond the intervention period. 

## 5. Conclusions

This study aimed to determine whether hydrotherapy influences behaviours which impact mental health and well-being in children with ASD. The findings of this randomised crossover-controlled pilot trial suggest that a once weekly, 4 week hydrotherapy (or water-based activity) intervention may positively influence behaviours related to the mental health and well-being of children with ASD in the immediate term. No negative effects were reported following the study; therefore, hydrotherapy may be a viable therapy option for children with ASD who present with emotional and behavioural concerns impacting their mental health and well-being. Whilst these findings cannot be directly generalized to all children with ASD, the findings of this study warrant larger intervention-based studies to explore the use of hydrotherapy for children with ASD across a variety of ages. Future studies may benefit from larger sample sizes, longer intervention periods to strengthen findings and should consider group versus individual sessions as a covariate which may impact the outcomes. Whilst hydrotherapy may be an appropriate therapeutic tool to use for children with ASD, one-on-one hydrotherapy sessions are recommended to minimise risk of injury in the water. Hydrotherapy may be appropriate to be used clinically, both independently and as an adjunct therapy, to enhance behaviours impacting mental health and well-being of children with ASD.

## Figures and Tables

**Table 1:** Participant characteristics by gender.

Participants	Mean Age (Years)	Gender	Receives Special Education	Academic or Other Problems at School	Neuro -Developmental Diagnoses	Adjunct Therapy
1–6	9.21	M	Yes (n = 6)	Yes (n = 5) No (n = 1)	ASD (n = 6) ADHD (n = 1	ST (n = 4) OT (n = 4) PT (n = 2) PSY (n = 1) TD (n = 1)
7–8	7.26	F	Yes (n = 1) No (n = 1)	Yes (n = 1) No (n = 1)	ASD (n = 2)	ST (n = 1) OT (n = 1)

ASD = Autism Spectrum Disorder; ADHD = Attention Deficit Hyperactivity Disorder; ST = Speech Therapy; OT = Occupational Therapy; PT = Physiotherapy; PSY = Psychology; and TD = Therapy dog.

**Table 2:** Target skills, activities and equipment for hydrotherapy interventions.

Skill	Activity
Cardiovascular Fitness	• Free swim * Swimming through large plastic hoop * Horizontal rope climbing with 10 m nautical rope Somersaulting in waterJumping into water from pool edge Prolonged independent submersion * Diving to place/retrieve objects from pool bottom: rubber animals, play coins, rings * Races: front crawl, back crawl *
Swimming Skills	• Free swim * Races: front crawl, back crawl * Swimming through large plastic hoop * Diving to place/retrieve objects from pool bottom: rubber animals, play coins, rings *
Relaxation and Sensory Input	• Weaving/seaweeding Dragging through water with plastic kickboard on stomach and side Floating on back Prolonged independent submersion * Dragging forward/backward and spinning in inflatable ring
Cognitive tasks	• Shape matching with Shape-O Counting play diving coins
Balance	• Walking along narrow beam in water Walking along submerged 10 m nautical tight rope Egg and spoon balance up/down entry ramp *
Eye–Hand Coordination	• Throwing/catching soft soccer ball Diving to place/retrieve objects from pool bottom: rubber animals, play coins, rings * Play foam sword fighting Retrieving floating rings with foam sword or wooden spoon Ring toss Hitting and keeping rubber ball above water in group Egg and spoon balance up/down entry ramp * Tossing small plastic balls into floating hoop

* Denotes an activity targeting multiple skills.

**Table 3:** Enjoyment scale results from hydrotherapy group interventions.

Participant	Intervention 1	Intervention 2	Intervention 3	Intervention 4
1	4	3	3	N/A
2	4	4	4	4
3	1	1	4	4
4	3	4	3	4
5	3	4	N/A	N/A
6	4	4	N/A	4
7	3	3	3	3
8	4	1	2	2

0 = no fun at all, 1 = boring, 2 = a bit of fun, 3 = fun, 4 = super fun, and N/A = child missed the hydrotherapy group session.

**Table 4:** Mean Child Behaviour Checklist percentile scores for groups 1 and 2 at baseline (week 0).

Domains and Syndromes	Groups 1 and 2 Combined Mean (SD)	Group 1 Mean (SD)	Group 2 Mean (SD)
Internalising Problems Domain			
Anxious/Depressed Syndrome	83.00 (18.38)	90.75 (9.84)	75.25 (23.06)
Withdrawn/Depressed Syndrome	75.63 (17.04)	73.00 (20.45)	78.25 (15.52)
Somatic Complaints Syndrome	75.38 (20.90)	81.75 (21.63)	69.00 (21.02)
Domain Summary	79.63 (24.32)	87.5 (15.26)	71.75 (31.33)
Externalising Problems Domain			
Rule Breaking Behaviour Syndrome	71.75 (19.20)	81.50 (15.61)	62.00 (19.04)
Aggressive Behaviour Syndrome	68.13 (16.53)	76.75 (17.56)	59.50 (11.44)
Domain Summary	62.63 (28.36)	79.00 (14.51)	46.25 (30.84)
Other Problems Domain			
Social Problems Syndrome	83.75 (10.33)	86.00 (10.42)	81.5 (11.27)
Thought Problems Syndrome	92.00 (11.71)	94.25 (5.85)	89.75 (16.50)
Attention Problems Syndrome	98.38 (0.92)	98.50 (1.00)	98.25 (0.96)
Total Problems	88.375 (11.48)	94.00 (8.72)	82.75 (12.12)

**Table 5:** Mean Child Behaviour Checklist outcomes (t scores) of hydrotherapy intervention and control periods for the combined groups 1 and 2.

Domains and Syndromes	Intervention Period				Control Period			
	Pre-Intervention n = 8	Post-Intervention n = 8	Change Score	Pre- to Post-Intervention Difference	Pre-Control n = 8	Post-Control n = 8	Change Score	Pre- to Post-Control Difference
	Mean (SD)	Mean (SD)	%	*p*-value	Mean (SD)	Mean (SD)	%	*p*-value
Internalising Problems Domain								
Anxious/Depressed Syndrome	62.75 (9.44)	54.88 (5.17)	7.87	0.02 * ^††† 1^	58.50 (9.72)	61.25 (9.51)	−2.75	0.31
Withdrawn/Depressed Syndrome	58.88 (7.20)	56.25 (7.05)	2.63	0.34	59.25 (11.96)	57.38 (7.07)	1.88	0.50
Somatic Complaints Syndrome	59.00 (8.60)	55.50 (7.03)	3.50	0.18	55.75 (7.40)	56.38 (7.35)	−0.63	0.56
Domain Summary	62.25 (7.74)	53.88 (8.06)	8.38	0.03 * ^††† 2^	57.00 (11.76)	59.75 (7.81)	−2.75	0.29
Externalising Problems Domain								
Rule Breaking Behaviour Syndrome	57.63 (7.62)	54.75 (7.32)	2.88	0.07	54.50 (6.65)	54.88 (6.42)	−0.38	0.84
Aggressive Behaviour Syndrome	56.50 (8.19)	55.63 (10.24)	0.875	0.59	54.50 (8.59)	55.50(9.35)	−1.00	0.51
Domain Summary	53.88 (12.33)	49.00 (14.21)	4.88	0.06	49.88 (11.36)	51.88 (11.99)	−2.00	0.37
Other Problems Domain								
Social Problems Syndrome	59.13 (5.77)	55.00 (5.45)	4.13	0.08	58.75 (6.11)	57.00 (3.78)	1.75	0.36
Thought Problems Syndrome	67.88 (7.00)	61.00 (9.62)	6.88	0.03 * ^††† 3^	63.63 (10.01)	65.25 (7.74)	−1.63	0.59
Attention Problems Syndrome	75.13 (9.88)	66.63 (12.60)	8.50	0.01 * ^†† 4^	67.63 (10.91)	69.63 (13.46)	−2.00	0.53
Total Problems	64.38 (6.09)	55.63 (10.22)	8.75	0.02 * ^††† 5^	58.25 (10.00)	60.50 (7.21)	−2.25	0.32

* Indicates a significant difference between pre- and post-intervention scores. ^†^ = small effect size; ^††^ = medium effect size; ^†††^ = large effect size. (Degrees of freedom) = t critical score: ^1^ t(7) = 2.936: ^2^ t(7) = 2.087: ^3^ t(7) = 2.787: ^4^ t(7) = 3.915: ^5^ t(7) = 3.068.
